# The references level of cadmium intake for renal dysfunction in a Chinese population

**DOI:** 10.1038/s41598-018-27411-3

**Published:** 2018-06-13

**Authors:** Xiao Chen, Zhongqiu Wang, Guoying Zhu, Xiaoqiang Ding, Taiyi Jin

**Affiliations:** 1 Department of Radiology, Affiliated Hospital of Nanjing University of Chinese Medicine, 155 Hanzhong road, Nanjing, 210029 China; 2 Department of Nephrology, Shanghai Key Laboratory of kidney and dialysis, Zhongshan Hospital Fudan University, 180 Fenglin road, Shanghai, 200032 China; 3 Institute of Radiation Medicine, Fudan University, 2094 Xietu road, Shanghai, 200032 China; 4 Department of Occupational Medicine, School of Public Health, Fudan University, 130 Dongan road, Shanghai, 200032 China

## Abstract

Recent several studies indicated that a more restrictive dietary intake guideline for cadmium should be made for sufficient health protection. In the present study, we showed the references level of food cadmium intake (FCd) and total cadmium intake (TCd) for renal dysfunction by using benchmark dose (BMD) approach. 342 subjects living in a control and a cadmium polluted area were included in this study. The FCd, TCd and cadmium in urine (UCd) and blood (BCd) were calculated or determined. Urinary β_2_Microglobulin (UBMG) was determined as indicator of renal function. The median FCd, TCd, UCd and BCd were 1.4 g, 1.4 g, 3.1 μg/g creatinine(cr) and 1.3 μg/L in control and 3.3 g, 3.6 g, 13.5 μg/g cr and 12.1 μg/L in polluted area. The 95% lower confidence bounds of BMD (BMDLs) of FCd for renal dysfunction were 1.36–1.55 g (BMR = 10%) and 0.88–1.11 g (BMR = 5%). The BMDLs of TCd were 1.29–1.46 g (BMR = 10%) and 0.73–0.95 g (BMR = 5%). FCd and TCd are valuable markers for the predication of renal dysfunction induced by cadmium. The BMDLs of FCd were close to previous report in Japan and the BMDLs of TCd were lower than the critical standard previously reported, in particular at BMR of 5% which can be interpreted as representing the influence of smoking.

## Introduction

Cadmium (Cd) that widely distributed in the environment is one of toxic heavy metals. It can enter the human bodies through the food chain. Cadmium in the humans has a long biological half-life, 10–30 years^1^. The kidneys are the main organs for Cd accumulation and are considered as the critical target organs of Cd toxicity. Previous studies have demonstrated that Cd in urine (UCd) and blood (BCd) are associated with renal dysfunction, in particular to renal tubular dysfunction^2–4^. The references level of UCd and BCd for renal dysfunction have been reported by using benchmark dose (BMD) approach^5–7^. However, only few studies in Japan have shown the references level of dietary or total Cd intake for renal dysfunction^8,9^. Cd contamination is reported in dozens of places in China^10,11^, and renal dysfunction has been observed in the residents living in those areas^5^. However, few studies have investigated the references level of Cd intake for renal dysfunction in China. In addition, the influence of smoking on the estimation of Cd references level has not been clarified.

The reference levels of UCd for renal dysfunction have been widely investigated. Thresholds of UCd, 2 μg/g creatinine for the onset of early renal damage^12–14^ to 10 μg/g creatinine for the occurrence of the classic tubular proteinuria^15^ have been reported. However, several studies have challenged the role of UCd as a potentially exposure biomarker^12,16–18^ because UCd is easily affected by physiological variations, such as diuresis. Dietary Cd intake might be a better marker than UCd^12^.

BMD method has been used for estimation of reference levels of UCd, BCd and dietary Cd intake^7,9,19,20^. In the current study, the reference levels of food Cd intake (FCd) and total Cd intake (TCd) for renal dysfunction were calculated by using BMD approach in Chinese population. Moreover, we also investigated the effect of smoking on the estimated reference level.

## Results

### Characteristics of study population

Firstly, we showed the characteristics of study population (Table 1). No significant differences were observed in age and gender between these two areas. The median FCd, TCd, UCd and BCd in control and polluted areas were 1.4 g and 3.3 g, 1.4 g and 3.6 g, 3.1 μg/g cr and 13.5 μg/g cr, 1.3 μg/L and 13.1 μg/L, respectively. Significant difference was found in the level of urinary β_2_Microglobulin (UBMG) between the polluted area and the control (0.3 mg/g cr vs 0.1 mg/g cr, p < 0.01).

**Table 1:** Characteristics of study population.

Variables	Control area (n = 123)	Polluted area (n = 219)	*p* Value
Age (ys)	45.6 ± 11.2	46.1 ± 11.4	>0.05
Male: female	62/61	107/112	>0.05
Smoking	54 (43.5)	90 (41.1)	>0.05
Food Cd Intake(g)	1.4 (0.7–1.9)	3.3 (2.3–3.7)	<0.01
Total Cd intake(g)	1.4 (0.8–2.1)	3.6 (2.4–6.2)	<0.01
UCd(μg/g cr)	3.1 (0.5–10.6)	13.5 (3.2–43.6)	<0.01
BCd(μg/L)	1.3 (0.5–4.7)	12.1 (4.4–38.7)	<0.01
UBMG(mg/g cr)	0.1 (0.03–0.74)	0.3 (0.05–4.6)	<0.01

UCd: cadmium in urine; BCd: cadmium in blood; UBMG: urinary β_2_Microglobulin

Age, weight and height are shown as mean value ± standard deviation.

Food Cd intake, total Cd intake, UCd, BCd, and UBMG are shown as median (5–95% percentile).

### The association between cumulative Cd intake and UCd, BCd and UBMG

**Figure 1:**
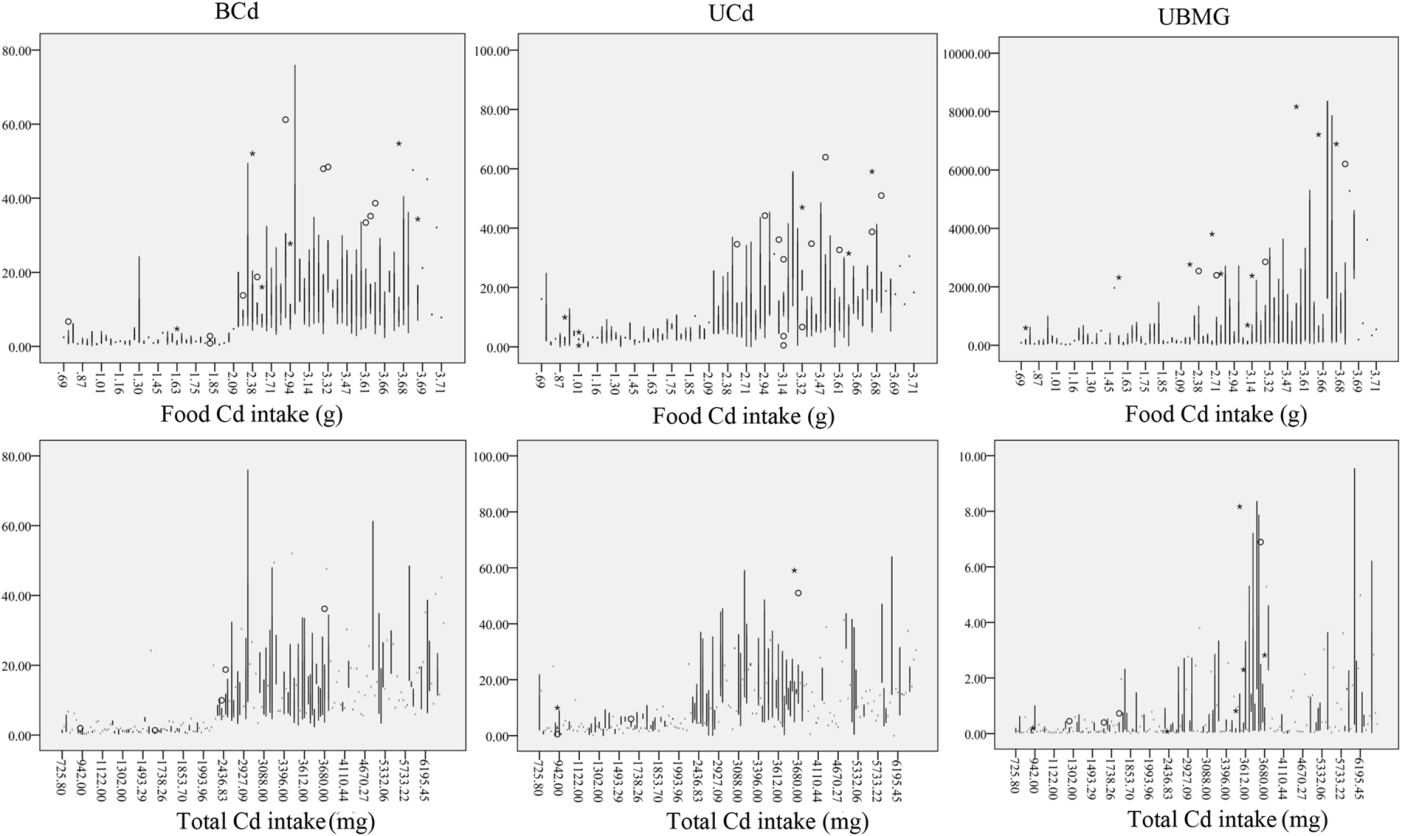
The association between blood cadmium (BCd), urinary cadmium (UCd) renal markers and food cadmium intake and total cadmium intake. The UCd, BCd and UBMG were all increased with the food cadmium intake or total Cd intake. UCd: cadmium in urine; BCd: cadmium in blood; UBMG: urinary β_2_Microglobulin

Next, we observed the association between FCd and UCd, BCd and UBMG (Fig. 1). The UCd, BCd and UBMG were all increased with the FCd. Similar result was found between TCd and UCd, BCd and UBMG.

**Figure 2:**
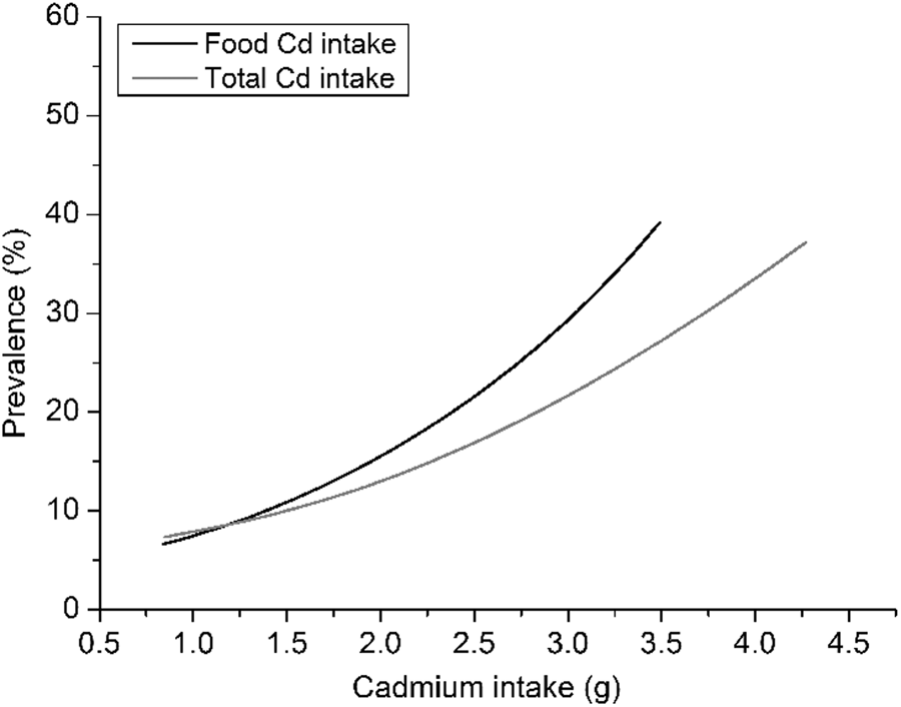
The association between food cadmium (Cd) intake, total Cd intake and urinary β_2_Microglobulin (UBMG). Renal dysfunction was increased upon increasing levels of cumulative Cd intake from food or total Cd intake.

Correlation analysis also showed that UBMG was positively correlated with the FCd, TCd, UCd and BCd (p < 0.05 or 0.01) (Fig. 2). The correlation between UBMG and FCd showed the highest correlation coefficient compared with UCd, BCd and TCd (0.44 vs 0.30, 0.37 and 0.39).

### Prevalence of renal dysfunction and Cd intake

**Figure 3:**
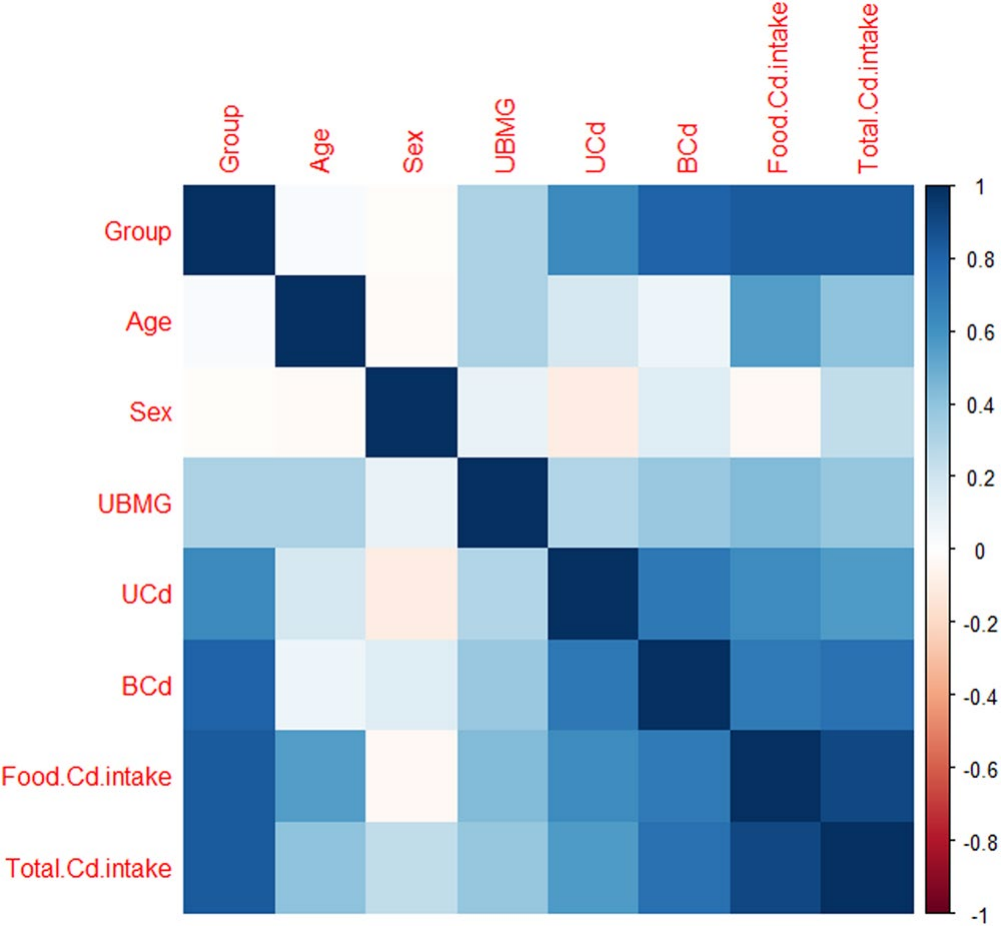
The correlation analysis between cadmium levels and urinary β_2_Microglobulin (UBMG). Food cadmium intake, total cadmium intake, urinary cadmium (UCd) and blood cadmium (BCd) were all positively associated with UBMG (p < 0.05 or 0.01).

Subsequently, we evaluated the association between the prevalence of renal dysfunction and Cd intake (Fig. 3). The data indicated that the prevalence of renal dysfunction increased upon increasing levels of FCd. The associations were statistically significant in the Chi-square for trend test (p < 0.01). Similar result was observed between TCd and renal dysfunction.

### The BMD of cumulative Cd intake for renal dysfunction

Subsequently, we calculated the BMD and their 95% lower confidence bounds (BMDLs) of FCd and TCd based on the elevated UBMG (Table 2). The mean cumulative FCd (0.84, 1.50, 2.60 and 3.49 g) and TCd (0.85, 1.55, 2.58 and 4.27 g) were used in the models to calculate the BMD and BMDL, respectively. The BMDLs of FCd were 1.36–1.55 g (BMR = 10%) and 0.88–1.11 g (BMR = 5%). The BMDLs of TCd were 1.29–1.46 g (BMR = 10%) and 0.73–0.95 g (BMR = 5%). A decrease of 0.15 g in BMDLs in TCd was observed compared with the FCd.

**Table 2:** Benchmark dose (BMD) and the 95% lower confidence limit of the benchmark dose (BMDL) of cadmium intake

	Model	BMR = 10%		BMR = 5%		p
		BMD	BMDL	BMD	BMDL	
Food Cd intake	LogLogistic	2.48	1.41	2.11	0.94	>0.1
	LogProbit	2.53	1.55	2.21	1.11	>0.1
	Gamma	2.50	1.36	2.15	0.88	>0.1
Total intake (food + smoking)	LogLogistic	2.96	1.35	2.44	0.79	>0.1
	LogProbit	2.95	1.46	2.53	0.95	>0.1
	Gamma	2.96	1.29	2.47	0.73	>0.1

P values were obtained from the chi-square test, with the Pearson goodness of fit test, if P > 0.05 then the equation is a good fit.

BMR: benchmark response.

The Cd concentration in tobacco in control area was significant lower than that in the polluted area (1.86 mg/kg vs 17.4 mg/kg) in our study. Next, we calculated the BMDLs of TCd hypothesizing that all the subjects smoke cigarettes containing similar concentration of Cd (1.86 mg/kg). Our data showed that no obvious decrease was observed in TCd compared with FCd (Supp Table 1).

Moreover, we also calculated the BMD and BMDLs of TCd in the smoking populations (Supp Table 2). The BMDLs of TCd were 1.09–1.20 g (BMR = 10%) and 0.52–0.66 g (BMR = 5%). The BMDLs were lower than those in total population.

### Receiver operating characteristic (ROC) analysis

**Figure 4:**
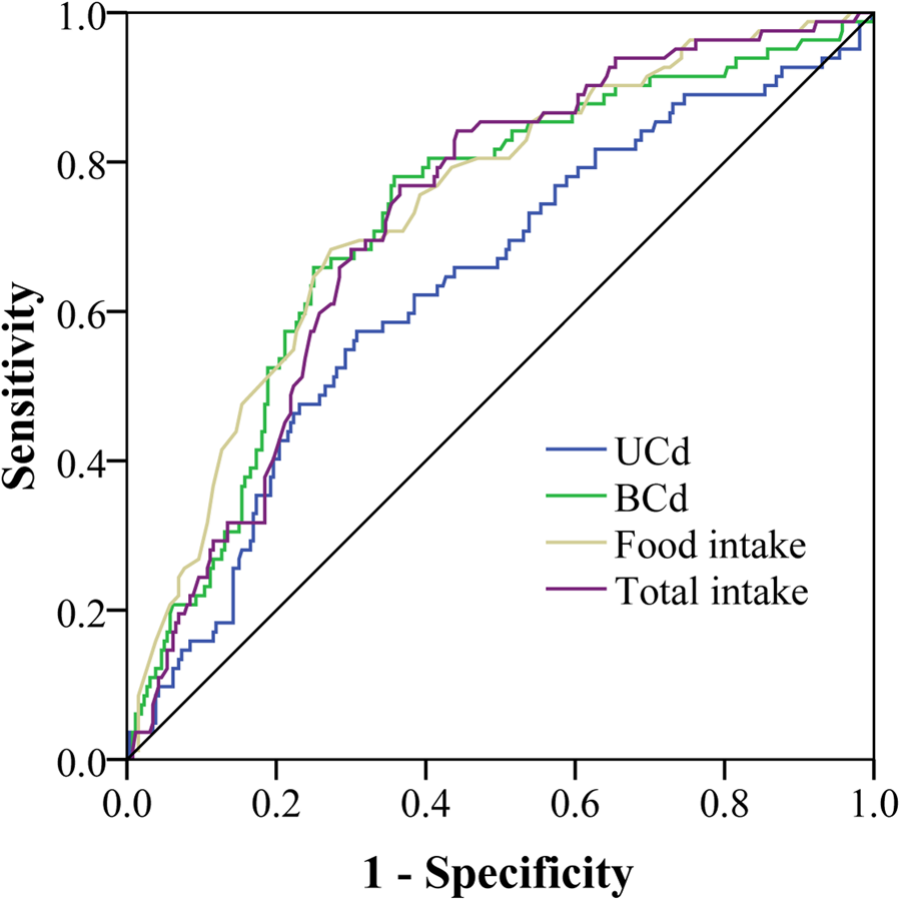
Receiver operating characteristics analysis of food cadmium intake, total cadmium intake, BCd and UCd in predicating renal dysfunction. BCd: cadmium in blood; UCd urinary cadmium.

Subsequently, we assessed the performance of FCd, TCd, UCd and BCd in predicating renal dysfunction (Fig. 4) by using ROC analysis. The area under curve (AUC) was 0.74 (95%CI: 0.68–0.80), 0.72 (95%CI: 0.67–0.78), 0.63 (95%CI: 0.56–0.70) and 0.72 (95%CI: 0.66–0.79) for FCd, TCd, UCd and BCd, respectively.

## Discussion

The reference levels of dietary Cd intake or total Cd intake for renal dysfunction have not been clarified. Several studies in Japan have reported the reference levels of lifetime Cd intake. Cd-contaminated food is one of major food safety issues in China^21^. However, no data have shown the tolerable levels FCd or TCd in China. In our study, the reference levels of FCd or TCd were calculated by using BMD approach. Our data showed that the BMDLs of FCd were close to previous report in Japan which considered the influence of drinking/cooking water^9^. However, our data was lower than other two studies in Japan^8,22^. In addition, our result also indicated that the BMDL of TCd was lower than the FCd. The difference of 0.15 g was due to the influence of smoking.

Previous several studies have shown the reference level of UCd for renal dysfunction in China^5,19^. However, the roles of UCd need careful reevaluation^18^. Dieresis and variation in urinary flow rates may affect UCd levels^12,17,18,23^. Cd intake from food has been suggested as a more useful biomarker than UCd^12^. Our data showed that FCd and TCd were all positively correlated with UCd, BCd and UBMG. In addition, FCd showed the highest correlation coefficient. ROC curve further indicated that the AUC of FCd was higher than UCd and BCd. Our data indicated that FCd is also a valuable marker that can reflect renal dysfunction.

Previous study showed that the threshold values of lifetime Cd intake were 1.0 g in men and 0.8 g in women if considering the water intake^9^. Our data of FCd was also included the Cd intake from water. The BMDLs of our study were 0.88–1.11 g which was very close to their data. Based on the previous data and our data, the tolerable daily intake was about 1.38 mg/month-1.67 mg/month for 50–60 years old person which was very close to the provisional tolerable monthly intake (PTMI) of 25 μg/kg body weight recommended by based on epidemiological studies (Joint FAO/WHO Expert Committee on Food Additives, JECFA 2010). However, the BMDLs of TCd were 0.73–0.95 g. For 50–60 years old person, the tolerable month intake was about 1.12–1.4 mg/month which was lower than PTMI recommended by JECFA. Moreover, for those smoking population, much lower BMDLs were obtained compared with the total population. If we did not consider the contribution of smoking, the PTMI recommended by JECFA may sufficiently protect against Cd-induced renal effect. However, if the influence of smoking was considered, a stricter tolerable level should be recommended. In addition, the decrease of BMDLs in smoking population also indicates that those populations are susceptible to renal dysfunction.

Based on the BMDLs obtained in the present study, the exposure levels for some persons living in control area were also relatively high. UBMG was also positively correlated with TCd or FCd in subjects living in control area. Our data is in accordance with the findings that environmental cadmium exposure can cause renal dysfunction in the general population^3,24^. For sufficient protection, a stricter dietary intake guideline should be established. A 25 μg/d (for 70 kg body weight) of PTWI was recommended by European Union^25^. In addition, if a more sensitive biomarker or earlier biomarker was used, such as urinary N-acetyl-β-D-glucosaminidase (UNAG), the calculated BMDLs may be much lower than the data above. Those data indicated that much effort should be made to control the maximally permissible concentration values for FCd as low as reasonably achievable^26^.

Cigarette smoking is another important exposure pathway of Cd in the general population. However, the contribution of Cd in tobacco was not considered in previous studies when calculate the BMDLs because the Cd uptake from smoking was small in subjects living in Cd polluted areas^22^. In our study, we also considered the contribution of smoke Cd intake to BMDL calculation. The BMDL of TCd decreased by 0.15 g compared with FCd which indicated that the tolerable levels should be much lower than the level recommended by JECFA if considered the contribution of smoking. However, the tobacco in polluted area contained a higher concentration of Cd than these in control or commercial cigarettes. Then we calculated the BMDL of total intake hypothesizing that the subjects in polluted area smoke the same cigarettes with control. The BMDL of total intake only slight decreased compared with food intake. Our data showed that the PTMI should be decreased by 1.4 μg/kg for a 60 kg of 50-year-old person. If the contribution of Cd intake from smoking to TCd was much lower than that from food, smoking may only slightly affect the BMDLs. However, if there are obviously differences in Cd intake from smoking, the contribution of smoke could not be neglected. We also estimated the BMDLs of TCd in the smoking subjects. The data indicated that a lower references level should be made for those smoking subjects than the total population.

Our study has several limitations. First, the influence of age on BMDL calculation was not considered because the small population sizes. Second, it has been shown that there are differences in BMDL between men and women. However, no such calculation was performed in our study due to the small population size in control. Third, many biomarkers such as UNAG, Kidney injury molecule-1 can reflect renal tubular function. Only UBMG was adopted in our study. Finally, the absorbed cadmium may be more closely related with renal dysfunction than FCd or TCd. However, we did not estimate the cadmium uptake because the absorption rate of cadmium varies with the individual. Consequently, we did not calculate the BMD or BMDLs of cadmium uptake in this study.

In conclusion, FCd is also a valuable marker for Cd-induced renal effects. The BMDLs of FCd for Cd-induced renal dysfunction was estimated to be 0.88–1.11 g (BMR = 5%) in Chinese population. The BMDLs of total Cd intake was estimated to be 0.73–0.95 g (BMR = 5%). The difference of 0.15 g was due to the influence of smoking.

## Materials and Methods

### Study area and population

The detailed information has been reported in our previous study^27^. Briefly, two areas, located in Jiangxi Province, China, were included. A polluted area, named Dayu, was rich in Tin and tungsten. The mining of Tin and tungsten was begun from AD 660 and 1908, respectively. The rice fields were polluted with the wastewater. An average Cd concentration was 0.59 mg/kg in unpolished rice^3^. The drinking water was also polluted by wastewater. The Cd levels in vegetables were 0.06–0.66 mg/kg^27^. The previous studies showed that the daily Cd intake was about 299 μg/d in women and 313 μg/d in men^28^. We chose a non-contaminated area nearby as a control area. The Cd in polished rice was less than 0.08 mg/kg. The Cd levels in vegetables were ranged from 0.02 to 0.14 mg/kg. The total Cd intakes were 52.5–55.0 μg/d in men and 50.7 μg/d in women. The rice was the staple food. The people living in the two areas have common living conditions and lifestyles. More detailed information about the study areas and population has been reported in previous studies^28,29^.

Total of 342 local residents (123 subjects in control and 219 subjects in polluted area), 173 women and 169 men, were finally included. Only the persons who have lived in the areas ≥25 years were included in this study. All participants completed two questionnaires. One was the weekly food consumption questionnaire, and the other was used for the collection of demographic information and smoking habits. All participants completed the informed consent. This study was proved by the Institutional Review Board of Fudan University, China, and all protocols were performed according to the approved guidelines. More detailed information could be found in the previous studies^28,29^.

### Cd intake estimation

The previous studies have given the detailed information about the Cd intake estimation^28,29^. Briefly, the questionnaire of weekly food consumption was adopted to collect the type and amount of main foods consumed. 60 households were randomly collected from five villages in polluted area and two villages in control area. We mixed each types of food in same area together for chemical analysis. Nineteen samples of local tobacco were also collected. Then, after dried and washed, samples were dissolved in nitric acid for Cd determination by using flame atomic absorption spectrometry (AAS) or graphite furnace AAS (Perkin Elme, Model PE-3030). The following equations were used for Cd intake analysis: FCd = Σ (Cd in food × food consumption) + Cd in water × water consumption; TCd = FCd + Cd in tobacco × tobacco consumption × 10%. For estimation of Cd intake from food, the following age-related weighting factor was used during the calculation: 0–9 years, 0.41; 10–19 years, 0.89; 20–59 years, 1.000; ≥60 years, 0.823^4,29^.

### Sample collection and cadmium analysis

The detailed information was described in our previous study^27^. Briefly, the collectors were immersed in HNO_3_ solution (5%) for 24 h, and then they were washed with deionized water and dried. The blood and urine samples were stored at −20 °C in local laboratory. UCd and BCd were determined by using flame AAS after wet digestion^27^. We also performed quality control during the Cd determination^28,30^.

### Renal markers determination

The detailed information was described in our previous study^27,28^. The level of UBMG was determined by using radioimmunoassay (RIA) kit. Urinary creatinine (UCr) was determined by using Jaffe reaction method^31^. UBMG were adjusted with UCr and showed as milligram/g cr (mg/g cr). The cut-off values of 0.8 mg/g cr were used according to the 95th percentile of UBMG in the control (0.75 mg/g cr). UBMG levels >0.8 mg/g cr were regarded as “elevated.” Values below 0.8 mg/g cr were considered as “normal.”

### Benchmark dose analysis

We use the benchmark dose software (BMDs, Version 2.6.01, Environmental Protection Agency, USA; https://www.epa.gov/bmds/what-benchmark-dose-software-bmds) to calculate the BMD and BMDLs. Benchmark response (BMR) of 5% and 10% were both used during the calculation. BMD and BMDLs were calculated using Gamma, Loglogistic and Logprobit models. We divided the subjects into four groups based on the FCd or TCd. Goodness of fit test was performed.

### Statistical analysis

SPSS 16.0 (SPSS Inc., Chicago, IL, USA) was used for statistical analysis. Data were listed as mean (data with normal distribution) or median (data with abnormal distribution). We used independent-samples t test or Mann-Whitney *U*-test or Chi-square test to compare the data between control and polluted area. The associations between exposure markers and renal effect biomarkers were evaluated by spearman correlation analysis. The performances of FCd, TCd, UCd and BCd in predicating renal dysfunction were evaluated by receiver operating characteristic (ROC) analysis. If P < 0.05 statistical significance was considered.

### Data availability

All data generated or analyzed during this study are included in this published article (and its Supplementary Information files).

## Electronic supplementary material

Supplementary Information
