# Supplementary material for: The references level of cadmium intake for renal dysfunction in a Chinese population
Source: Sci Rep. 2018 Jun 13;8:9011. doi: 10.1038/s41598-018-27411-3 (PMC5998016; doi:10.1038/s41598-018-27411-3)
Supplement: Supplementary file 1 — Supplementary Information [file 41598_2018_27411_MOESM1_ESM.doc]

**The references level of cadmium intake for renal dysfunction in a Chinese population**

Xiao Chen^1,2^, Zhongqiu Wang^1^, Guoying Zhu^3^, Xiaoqiang Ding^2^, Taiyi Jin^4^

1 Department of Radiology, Affiliated Hospital of Nanjing University of Chinese Medicine, 155 Hanzhong road, Nanjing 210029, China

2 Department of Nephrology, Shanghai Key Laboratory of kidney and dialysis, Zhongshan Hospital Fudan University, 180 Fenglin road, Shanghai 200032, China

3 Institute of Radiation Medicine, Fudan University, 2094 Xietu road, Shanghai 200032, China

4 Department of Occupational Medicine, School of Public Health, Fudan University, 130 Dongan road, Shanghai 200032, China

Corresponding author: Xiaoqiang Ding, Department of Nephrology, Zhongshan Hospital Fudan University, 180 Fenglin road, Shanghai 200032, China, email: ding_xiaoqiang@zs-hospital.sh.cn; Taiyi Jin, Department of Occupational Medicine, School of Public Health, Fudan University, 130 Dongan road, Shanghai 200032, China, email: tyjin@shmu.edu.cn

Supplemental data

Table 1 Benchmark dose (BMD) and the 95%lower confidence limit of the benchmark dose (BMDL) of food cadmium intake and total cadmium intake

|  | Model | BMR=10% | | BMR=5% | | p |
| --- | --- | --- | --- | --- | --- | --- |
|  |  | BMD | BMDL | BMD | BMDL |  |
| Food Cd intake | LogLogistic | 2.48 | 1.41 | 2.11 | 0.94 | >0.1 |
|  | LogProbit | 2.53 | 1.55 | 2.21 | 1.11 | >0.1 |
|  | Gamma | 2.50 | 1.36 | 2.15 | 0.88 | >0.1 |
| Total Cd intake | LogLogistic | 2.52 | 1.39 | 2.11 | 0.92 | >0.1 |
|  | LogProbit | 2.56 | 1.53 | 2.22 | 1.10 | >0.1 |
|  | Gamma | 2.54 | 1.36 | 2.16 | 0.91 | >0.1 |

The total cadmium intake includes food intake and smoking intake. In this calculation, the smoking intake was estimated based on the tobacco in control area. The mean total cadmium intakes (0.85, 1.52, 2.56 and 3.59 g) were used in the models.

P values were obtained from the chi-square test, with the Pearson goodness of fit test, if P>0.05 then the equation is a good fit.

UBMG: urinary β_2_Micorgloblin
